# Sensing of Pyrophosphate Metabolites by Vγ9Vδ2 T Cells

**DOI:** 10.3389/fimmu.2014.00688

**Published:** 2015-01-22

**Authors:** Siyi Gu, Wioletta Nawrocka, Erin J. Adams

**Affiliations:** ^1^Department of Biochemistry and Molecular Biology, University of Chicago, Chicago, IL, USA; ^2^Committee on Immunology, University of Chicago, Chicago, IL, USA; ^3^Committee on Cancer Biology, University of Chicago, Chicago, IL, USA

**Keywords:** Vγ9Vδ2, phosphoantigens, T cells, T cell receptor, butyrophilins, B30.2

## Abstract

The predominant population of γδ T cells in human blood express a T cell receptor (TCR) composed of a Vγ9 (Vγ2 in an alternate nomenclature) and Vδ2 domains. These cells came into the limelight when it was discovered they can respond to certain microbial infections and tumorigenic cells through the detection of small, pyrophosphate containing organic molecules collectively called “phosphoantigens” or “pAgs.” These molecules are intermediates in both eukaryotic and prokaryotic metabolic pathways. Chemical variants of these intermediates have been used in the clinic to treat a range of different cancers, however, directed optimization of these molecules requires a full understanding of their mechanism of action on target cells. We and others have identified a subclass of butyrophilin-related molecules (BTN3A1-3) that are directly involved in pAg sensing in the target cell, leading to engagement and activation of the T cell through the TCR. Our data and that of others support the pAg binding site to be the intracellular B30.2 domain of BTN3A1, which is the only isoform capable of mediating pAg-dependent stimulation of Vγ9Vδ2 T cells. Here, we review the data demonstrating pAg binding to the B30.2 domain and our studies of the structural conformations of the BTN3A extracellular domains. Finally, we synthesize a model linking binding of pAg to the intracellular domain with T cell detection via the extracellular domains in an “inside-out” signaling mechanism of the type characterized first for integrin molecule signaling. We also explore the role of Vγ9Vδ2 TCR variability in the CDR3 γ and δ loops and how this may modulate Vγ9Vδ2 cells as a population in surveillance of human health and disease.

Gamma delta T cells represent a conundrum when trying to understand the mechanisms of T cell ligand recognition that results in T cell activation. T cells expressing αβ T cell receptors (TCRs) that develop normally in the thymus recognize all antigens with the requirement of an antigen-presenting molecule belonging to the MHC superfamily. This MHC requirement encompasses conventional αβ T cell recognition of classical class I and class II MHC molecules as well as innate-like or semi-invariant T cell recognition of non-classical or MHC-like molecules. Examples include Type I invariant Natural Killer T (iNKT) and Type 2 NKT cell recognition of CD1d, Mucosal Associated Invariant T (MAIT) cell recognition of MR1, and non-conventional αβ T cell recognition of the human Group 1 CD1s (1). While these different T cell types recognize their various MHC ligands with diverse footprints, the fact remains that they are all “restricted” to recognizing antigens in the context of their respective MHCs.

This same MHC requirement does not appear to hold true for γδ T cells. While defining ubiquitous antigens for this lineage of T cells have been challenging, a clearer perspective has started to emerge with recent breakthroughs in antigen definition for these cells. While a comprehensive survey of these results is not the focus of this review and has been discussed elsewhere (2, 3), what has emerged from these studies is that γδ T cell are specific for both MHC and non-MHC proteins. To first understand this conundrum it is important to emphasize that γδ T cells cannot be grouped together as a whole. Instead, γδ T cells are divided into many different populations with different antigen reactivities, effector functions, and tissue residence (4, 5). Another important point is that there is little, if any, homology between γδ T cell populations in mice with those in humans, suggesting that these cells have rapidly adapted to different antigenic stimuli or immunological environments in the two different hosts. This is supported by the observed rapid evolution of many of the Vγ genes within the primate lineage (6, 7). Recent work from our laboratory has focused on two very different γδ T cell populations in humans: first, those that do recognize antigens in the context of CD1 molecules, which has been reviewed elsewhere (8); and secondly a population that appears to be MHC-independent and instead responds to small pyrophosphate antigens called “phosphoantigens (pAgs)”. This second population, called “Vγ9Vδ2”, “Vγ2Vδ2”, or “γ2δ2” by different groups, called Vγ9Vδ2 here, is the topic of this review. Recent breakthroughs by several groups have started to reveal the complex mechanism behind pAg regulation of this cell population. These findings have led to a shift in the paradigm of what specificities regulate T cell activity and a better understanding of the molecular mechanisms behind regulation of this important T cell population in humans.

Vγ9Vδ2 T cells are the major subset of γδ T cells found in human blood, comprising up to 5% of the T cells in healthy individuals and expanding to 20–50% during infection or disease (9). These cells play important roles in mediating immunity against microbial pathogens, including *Mycobacterium tuberculosis* and *Mycobacterium leprae* [the causative agents of tuberculosis and leprosy, respectively, reviewed in Ref. (10)], and can respond potently against certain types of tumor cells (11, 12). No homologous pAg-reactive Vγ9Vδ2 T cell population has been identified in rodents or lagomorphs, however, genes homologous to both Vδ2 and Vγ9 have been identified in other placental mammalian species including sloth, armadillo, lemur, aye aye, bottlenose dolphin, killer whales, and horse (13). Furthermore, expression of Vγ9Vδ2 TCRs was demonstrated in alpacas. This suggests that Vγ9Vδ2 T cells are present in species outside the primate lineage and likely predate the split of the placental mammals. The lack of Vγ9Vδ2 T cells in rodents and lagomorphs demonstrate that this lineage has been lost in some species, perhaps compensated by selection for alternative T cell subtypes.

As mentioned above, Vγ9Vδ2 T cells represent an important departure from the classical T cell recognition paradigm, in that no MHC or MHC-like molecules have been implicated in their activation (14). Instead, the aforementioned pAgs (Figure 1), which are pyrophosphate containing metabolites, are the key trigger (15–18). Amongst these, isopentenyl pyrophosphate (IPP) (16, 19) is generated from the endogenous mevalonate (MVA) pathway (HMG-CoA, cholesterol biosynthesis) and accumulates intracellularly during dysregulated metabolism in many types of tumor cells. Addition of aminobisphosphonates like zoledronate (NBP) or alkylamines also causes intracellular IPP accumulation through inhibition of farnesyl pyrophosphate synthase (12, 20, 21); this strategy is used frequently in studies of Vγ9Vδ2 T cell stimulation. A much more potent set of pAgs (i.e., HDMAPP/HMBPP: hydroxy-methyl-butyl-pyrophosphate) are microbial metabolites from the isoprenoid pathway (17) and represents “non-self” pathogen signals. A synthetic pAg, bromohydrin pyrophosphate (BrHPP) also strongly activates Vγ9Vδ2 T cells and is often used in *in vitro* functional experiments (22). The pAg-induced recognition of target cells is TCR dependent, as Vγ9Vδ2 TCR transfected Jurkat cells become activated by pAgs (23). While no direct interaction has been detected between pAgs and the γδ TCR, cell-to-cell contact is necessary in pAg-induced γδ T cell activation (14, 24), indicating that molecules expressed on the cell-surface of target cells or γδ T cells are required for activation.

**Figure 1:**
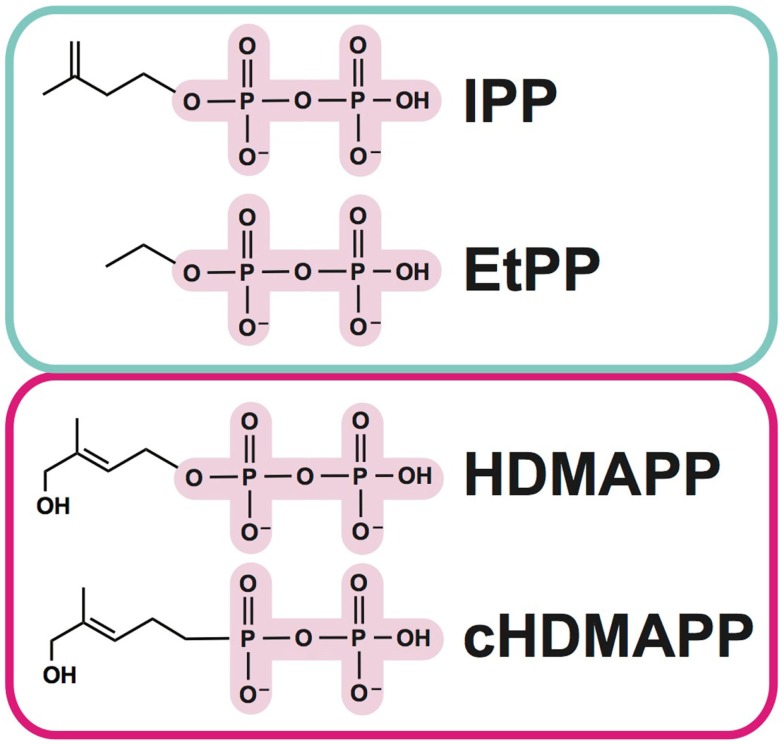
**Examples of phosphoantigens (pAgs) that stimulate Vγ9Vδ2 T cells**. Phosphoantigens (pAgs) that derive from the mevalonate pathway (“endogenous” IPP and synthetic derivative EtPP) are circled in blue whereas those that produced in the isoprenoid pathway of microbes (“exogenous” HDMAPP or synthetic derivative cHDMAPP) are circled in pink. The pyrophosphate motif is highlighted in pink; the chemically diverse organic moieties are shown as lines.

## Focus on Butyrophilins as Key Players in the Vγ9Vδ2 T Cell Response to pAgs

**Figure 2:**
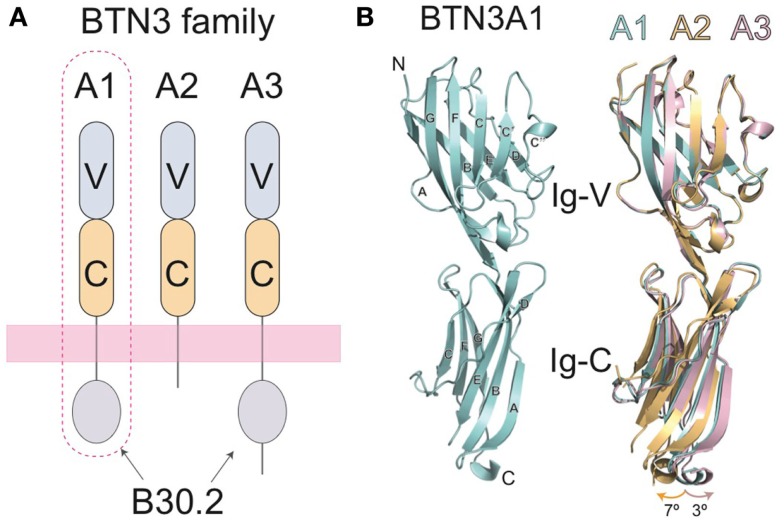
**Domain organization of the butryophilin-3 (BTN3) proteins**. **(A)** The BTN3A extracellular domains are members of the B7-superfamily with a membrane-proximal IgC domain and an N-terminal IgV domain. The extracellular domains between the three isoforms are highly similar, but are linked, via a single-pass transmembrane region, to intracellular domains that vary amongst the three BTN3A family members. BTN3A1 and BTN3A3 both contain intracellular B30.2 domains whereas BTN3A2 does not. BTN3A1, circled with a dotted line, has been shown to be necessary for pAg-induced activation of Vγ9Vδ2 T cells. **(B)** The three-dimensional structures of the extracellular domain of BTN3A1 is shown in cyan (left) and superimposed with the A2 and A3 isoforms (right) shown in gold and pink, respectively. The structures are highly homologous, with only small variations in the hinge angles between the IgV and Ig-C domains.

A major breakthrough in our understanding of Vγ9Vδ2 T cell activation came with the identification of the butyrophilin-3 (BTN3) protein family as a key mediator in this process (25). BTN3 proteins, also known as CD277, are type I membrane proteins with two immunoglobulin (Ig)-like extracellular domains (IgV and IgC) (26, 27) (Figure 2A) with close structural homology to the B7-superfamily of proteins. BTN3A molecules are members of a much larger butyrophilin superfamily with diverse roles in host homeostasis (28, 29). A key factor in the initial discovery of the role of BTN3A in Vγ9Vδ2 activation was the serendipitous discovery that a mouse antibody (clone 20.1), raised against human BTN3A molecules, caused a surprising proliferation and expansion of γδ T cells in IL-2 supplemented peripheral blood mononuclear cell (PBMC) cultures (25). The presence of this antibody elicited production of IFN-γ, TNF-α, and upregulation of the activation marker CD69 and has recently been shown to elicit very similar intracellular signaling in Vγ9Vδ2 T cells as pAgs (30). This phenomenon was restricted to the Vγ9Vδ2 population in PBMCs, with no effect on αβ T cells or those γδs not expressing a Vγ9Vδ2 TCR. Consistent with the lack of MHC requirement for stimulation of this γδ T cell population, addition of the 20.1 antibody to a panel of human tumor/transformed cell lines, some of which lack MHC surface expression, induced potent activation of responding Vγ9Vδ2 T cells (25). To rule out an effect of BTN3A expressed on Vγ9Vδ2 T cells, murine Vγ9Vδ2 TCR transductants, which do not express BTN3A molecules, were used as effector cells and shown to also respond to these 20.1 treated target cells. This experiment also confirms the requirement for the Vγ9Vδ2 TCR, supporting previous studies in Vγ9Vδ2 Jurkat transfectants (23). Similar results with the 20.1 Ab were found by another group (31). Other approaches also confirmed the role of BTN3A molecules in Vγ9Vδ2 T cell activation; taking a genetic approach, Vavassori et al. mapped genetic elements required for Vγ9Vδ2 T cell activation to the 3- to 27.4-Mb interval of human chromosome 6 and further refined their candidates by screening only those coding regions that had a predicted transmembrane element (32). Included within these candidates were the BTN3A molecules.

Three isoforms of BTN3A are present in humans, BTN3A1, BTN3A2, and BTN3A3, each encoded by a separate gene (26). The extracellular domains of the BTN3A molecules are highly sequence and structurally homologous, with only minor variations observed in the hinge angle between the IgV and IgC domains of their crystal structures when the three extracellular domain structures are superimposed (27) (Figure 2B). All three BTN3A isoforms are recognized by the 20.1 antibody and can mediate 20.1 mAb-induced activation of Vγ9Vδ2 T cells (25, 27), suggesting that a shared epitope on BTN3A molecules is involved in the process of Vγ9Vδ2 stimulation. Curiously, a different BTN3A specific antibody, 103.2, had an antagonistic effect on pAg-mediated Vγ9Vδ2 stimulation after addition to target cells, suggesting that it either blocks an epitope on the BTN3A extracellular domain or induces or stabilizes a non-stimulatory conformation of BTN3A on the cell-surface (25).

**Figure 3:**
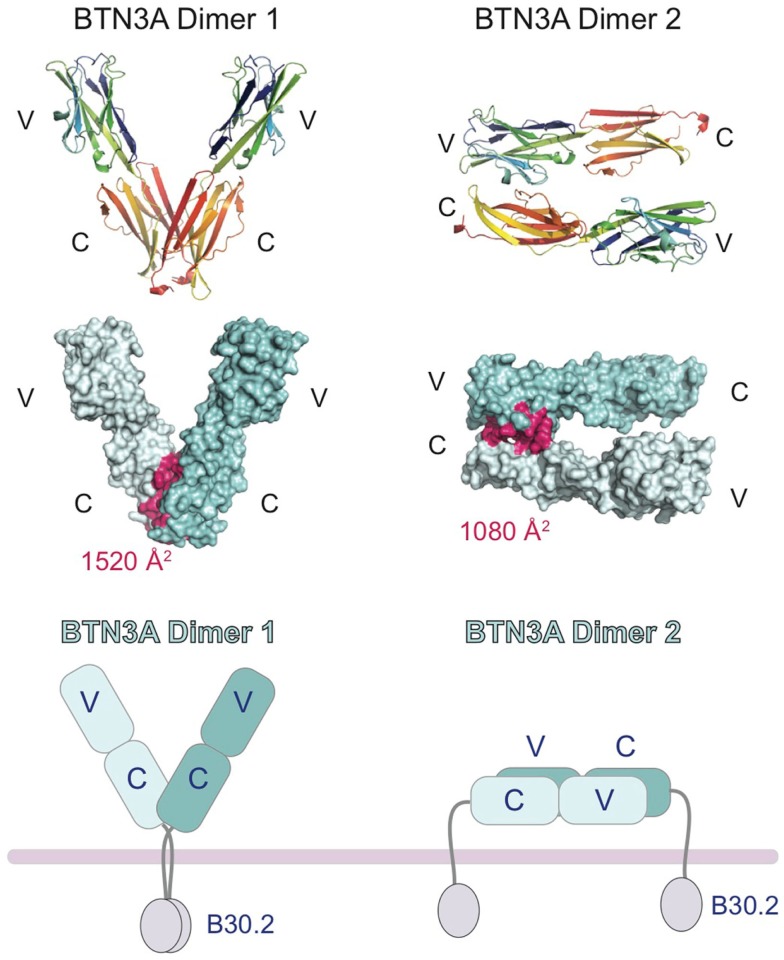
**Cartoon representation of the domain organization of the butryophilin-3 (BTN3) proteins**. Structures of the extracellular domains of the BTN3A1 proteins shown in the two dimeric states present in the crystal lattice. Dimer 1 (left) associates via the IgC domains and forms a V-shaped dimer, placing the intracellular B30.2 domains in close proximity to each other. Dimer 2 (right) associates in an head-to-tail fashion with the IgV domain of one BTN3A monomer interacting with the IgC domain of another. This would result in the dimer laying parallel to the cell-surface, with the intracellular B30.2 domains separated. The interface contact residues are colored pink and shown on the surface representation of the two dimeric forms (middle panel). The buried surface area (BSA) is shown for both dimers.

In the crystal structures of the BTN3A extracellular domains, two dimeric interfaces were observed (27), one that would generate a symmetric V-shaped homodimer positioning the C-terminal transmembrane domains close together (Dimer 1, Figure 3) and the other a head-to-tail homodimer with an asymmetric dimer interface, requiring the BTN3A molecules to lay flat, parallel to the cell-surface (Dimer 2, Figure 3). Both dimer interfaces were of appreciable size, Dimer 1 buried ∼1520 Å^2^ whereas Dimer 2 buried ∼1080 Å^2^. Both dimer interfaces were also highly conserved between the three BTN3A isoforms; only 2 out of the 18 interface residues in Dimer 2 differed between the BTN3A isoforms. However, the Dimer 2 interface was observed in the crystal structures of all three BTN3A isoforms indicating these differences were tolerated. Residues involved in the Dimer 1 interface differed at three positions across the three BTN3A isoforms although examination of the contacts in this interface revealed that these interactions involved only main chain atoms, thus tolerating variation in the composition of the side chain residues. This suggests that these extracellular domains can form heterodimers adopting both dimeric conformations when co-expressed on the cell-surface. Using soluble extracellular domains, we were able to establish that BTN3A molecules exist as stable homodimers in solution and, using a FRET approach, that the dimer conformation in solution was Dimer 1 (27). This does not, however, rule out the possibility that both dimers can exist on the cell-surface, perhaps stabilized through the transmembrane or intracellular domains not present in the soluble molecules.

**Figure 4:**
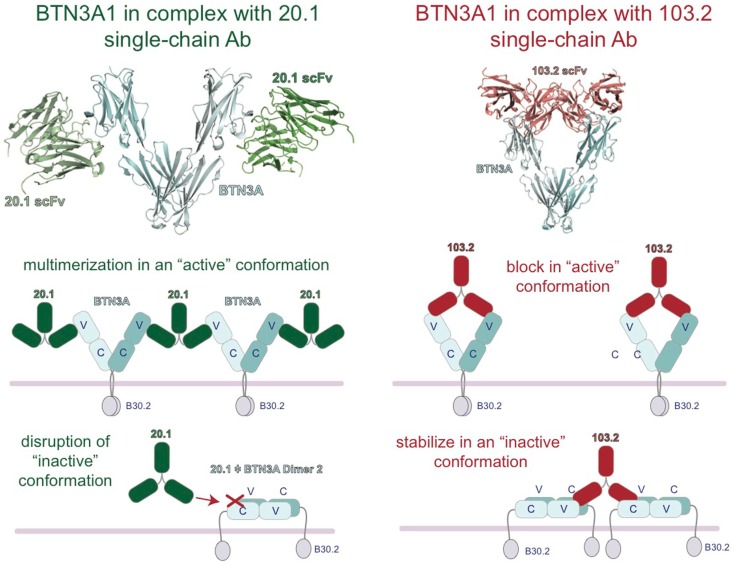
**Model of the regulation of BTN3A architecture by the agonist 20.1 and antagonist 103.2 antibodies**. Structures of the extracellular domains of the BTN3A proteins (cyan) in complex with agonist (20.1, green) and antagonist (103.2, red) antibody single chains (scFv). The 20.1 antibody cannot “reach” across a BTN3A dimer 1 to occupy both binding sites and therefore is likely to multimerize BTN3A molecules on the cell-surface (left). The 20.1 antibody binds to the Dimer 2 interface of the IgV domain and therefore would disrupt the Dimer 2 conformation on the cell-surface. The 103.2 antibody can bind both Dimer 1 and Dimer 2 conformations, either potentially blocking the activating Dimer 1 form or stabilizing the “inactive” Dimer 2 form on the cell-surface.

Insight into the binding sites and mode of action of the 20.1 and 103.2 antibodies was revealed with the complex crystal structures of single-chain versions of these antibodies (containing just the antigen-binding V domains) in complex with BTN3A1 (27). These complex structures demonstrated that these two antibodies bind to separate epitopes on the BTN3A surface (Figure 4), a result confirmed by competition-binding assays performed by Surface Plasmon Resonance (SPR). Curiously, the 20.1 antibody binding site positions the antibody such that it cannot bind bivalently to one BTN3A dimer as the two binding sites are too distant. For both 20.1 antibody binding sites to be occupied in the Dimer 1 conformation would require engagement of two separate BTN3A homodimers. Thus, binding of the 20.1 antibody could effectively cross-link these molecules on the cell-surface. Also interesting was the finding that the 20.1 binding site overlaps with that of the Dimer 2 interface, suggesting that binding of the 20.1 antibody would compete with the Dimer 2 conformation (Figure 4) and instead select for, and stabilize, the Dimer 1 conformation. The 103.2 epitope is accessible in both Dimer 1 and Dimer 2 conformations; in contrast to the 20.1 antibody, 103.2 would likely bind with both binding sites to one BTN3A Dimer 1, but would have to cross-link BTN3A molecules in the Dimer 2 conformation.

These results lead us to propose a model whereby these two dimeric states are related to the stimulatory potential of the cell upon which they are expressed. In normal, non-stimulatory conditions, BTN3A molecules would exist in the Dimer 2 state (head-to-tail) and thus not be in a state to provide a stimulatory signal to surveying Vγ9Vδ2 T cells. Upon addition of the 20.1 antibody, BTN3A molecules in the Dimer 2 conformation would be converted to Dimer 1; these would be cross-linked on the cell-surface via binding of one 20.1 antibody to two BTN3A dimers, and thus be converted into a “stimulatory” conformation permissible to stimulate Vγ9Vδ2 cells (Figure 4). The potential ability of the 20.1 antibody to cross-link BTN3A molecules in this model is consistent with the observed immobilization of BTN3A molecules via Flourescence Recovery after Photobleaching (FRAP) that occurs during conversion of a cell from a non-stimulatory to stimulatory state (25). This model also proposes that addition of 103.2 antibody could either block a site on BTN3A required for Vγ9Vδ2 cells activation or stabilize the Dimer 2 conformation on the cell-surface (Figure 4), thus leading to the inhibitory activity observed when this antibody is added in conjunction with pAg.

But what is the role of pAg in this process? Failed efforts to show a direct interaction between the Vγ9Vδ2 TCR and pAg early on suggested additional players were involved in this process; the requirement of cell–cell contact for Vγ9Vδ2 T cell stimulation also supported this hypothesis (14). Based on recent published results, two general models have been proposed to explain how pAg functions to stimulate Vγ9Vδ2 T cells. The first model is tantalizingly simple; it describes the extracellular domain of BTN3A molecules as “antigen-presenting” whereby BTN3A molecules associate with pAg and “present” it directly to the Vγ9Vδ2 TCR (32). While this model would fit well with the requirement of an antigen-presenting molecule for αβ T cell recognition of antigen, this model has met with controversy and is not supported by data generated from several groups and discussed further below. Model 2 is based on the finding that only one of the three BTN3A isoforms (BTN3A1) can support pAg-mediated Vγ9Vδ2 activation. This was demonstrated through siRNA knock-down experiments and reintroduction of individual BTN3A1, BTN3A2, or BTN3A3 isoforms; BTN3A1 alone was found to be pAg-reactive (25). This suggests that there is a unique element to this isoform that alone can initiate stimulation in a pAg specific way. Domain deletion and swapping experiments gave the first indication of the identity of this unique element: BTN3A1 lacking its intracellular domain failed to mediate pAg-mediated Vγ9Vδ2 stimulation but was highly stimulatory upon addition of the 20.1 antibody. BTN3A3, which cannot support pAg-mediated stimulation of Vγ9Vδ2 T cells, was made pAg stimulatory by swapping of its intracellular domain with that of A1 (31, 33). These data strongly support a pivotal role of the intracellular domain of the BTN3A1 isoform in pAg-mediated Vγ9Vδ2 stimulation. Model 2 is based on these findings and focuses on the intracellular domain of BTN3A1 as the pAg sensor.

**Figure 5:**
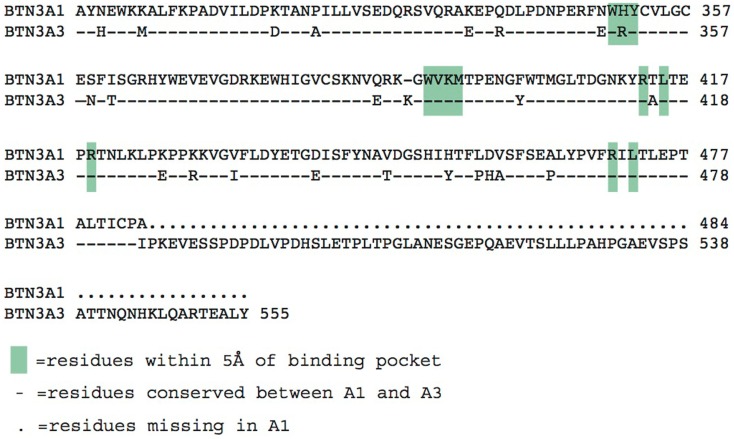
**Alignment of the intracellular B30.2 domains from BTN3A1 and BTN3A3**. Amino acids are shown in the single letter designations, BTN3A1 is the consensus. “-” indicates positions of identity with BTN3A1, differences are shown in their single letter abbreviations. Green boxes indicate residues within 5 Å of the phosphoantigen binding pocket. BTN3A3 has an additional polypeptide extension.

The three BTN3A molecules differ substantially in their intracellular domains; A1 and A3 each contain a B30.2 domain (also known as PRY/SPRY domains) whereas A2 lacks this domain (Figure 2). The B30.2 domains found in A1 and A3 are highly homologous, with 87% amino acid identity between the two (Figure 5). The intracellular region of A3, however, has a unique 70 amino acid extension C-terminal to its B30.2 domain (Figures 2 and 5). B30.2 domains are classified as protein–protein interaction domains and are found in other butyrophilin family members as well as non-related proteins (over 50 genes in the human genome have predicted B30.2 domains). Many B30.2 domain-containing proteins have been reported to be important in immune function, including the TRIM and pyrin families (34), although in most cases the binding partners have not been characterized. The importance of the B30.2 domain in pAg sensing was first demonstrated through swapping of just this domain between the A1 (capable of pAg activation) and A3 (incapable of activation) isoforms (33). Introduction of the A1 B30.2 domain into the A3 isoform converted this isoform to stimulatory for Vγ9Vδ2 T cell in the presence of pAg, whereas, the reverse swap (A3-B30.2 into A1 isoform) abrogated its ability to stimulate Vγ9Vδ2 T cells in a pAg-dependent fashion.

## Intracellular B30.2 Domain of BTN3A1 as the pAg Sensor

Direct interactions between both endogenous and exogenous pAgs with the B30.2 domain of BTN3A1 were measured with a highly sensitive technique called Isothermal Titration Calorimetry (ITC), which measures the heat absorbed or lost during binding events (33, 35). The affinities calculated from these techniques (KD = ∼1 μM for exogenous pAg, ∼1mM for endogenous pAg) also reflected the functional potency of these compounds in mediating stimulation of Vγ9Vδ2 T cells. The endogenous IPP pAg is typically 1,000-fold weaker potency than that of the exogenous HMBPP (36). Association studies with the HMBPP pAg were also shown via chemical shift perturbations (CSP) via Nuclear Magnetic Resonance (NMR), an equally sensitive technique (35). Of note, no association of pAgs could be measured with the BTN3A3-B30.2 domain, or to the extracellular domains of BTN3A1, A2 or A3, with either of these techniques (33, 35).

**Figure 6:**
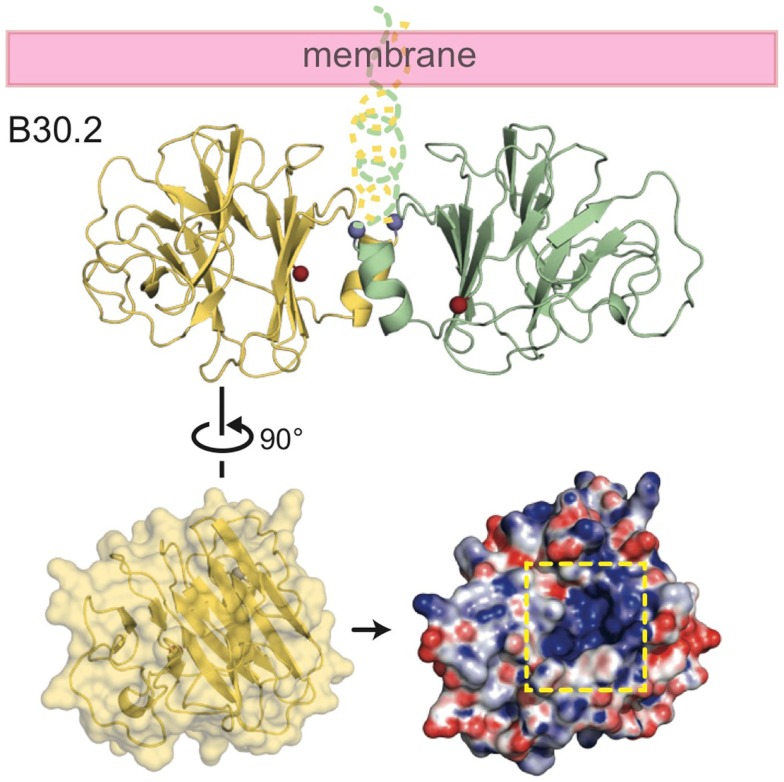
**Structure of the intracellular B30.2 domain of BTN3A1**. Shown is a cartoon diagram of the B30.2 domain dimer identified in the crystal lattice. Monomer 1 is shown in yellow, monomer 2 in green. N- and C-termini are shown as blue and red spheres, respectively, as is the putative orientation of these molecules in relation to the cell membrane. Turning monomer 1 approximately 90° and generating an electrostatic representation of the B30.2 surface, a highly positively charged pocket is clear (indicated by the dashed yellow box).

**Figure 7:**
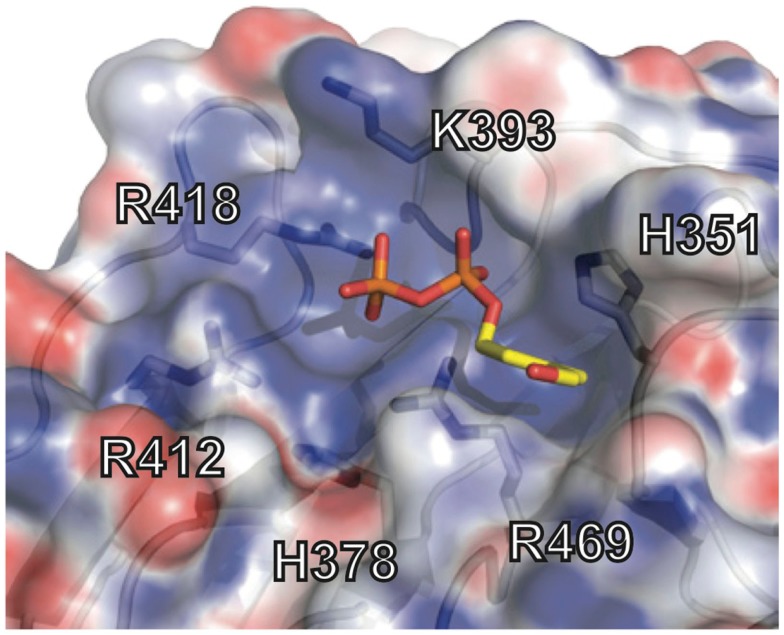
**Phosphoantigen binding pocket of B30.2 domain**. Close-up view of the B30.2 pAg binding pocket with the side chains lining the pocket shown under the semi-transparent surface. The positions are labeled with the numbering relative to the full-length BTN3A1 molecule. The pAg is shown as sticks, modeled into the binding pocket; phosphates are colored orange and red (oxygen) and the organic moiety is shown in yellow.

The crystal structure of the B30.2 domain of BTN3A1 (Figure 6) was highly informative in deciphering the pAg binding site (33). The structure of BTN3A1 B30.2 domain was highly homologous to previously reported B30.2 domains, in particular the B30.2 domain of Trim21, an intracellular Fc receptor (37). Importantly, specific to the BTN3A1 B30.2 domain was a highly positively-charged (basic) pocket nestled in Sheet A of the structure (Figure 6). This pocket was lined with basic residues including arginines (R412, R418, and R469), histidines (H351 and H378) and a lysine (K393) (Figure 7). The charge complementarity between the B30.2 positively charged pocket and the negative charge of pAgs made this an excellent candidate for pAg binding.

Charge swapping mutagenesis studies, where the basic residues were mutated to acidic (negatively charged), completely abrogated pAg binding and reactivity in cell stimulation assays, providing compelling evidence that this indeed was the pAg binding pocket (33). However, these results did not explain entirely the differences of pAg binding to the A1 versus A3-B30.2 domains. Close examination of the amino acid differences between these isoforms revealed a single amino acid difference that lay within the binding pocket: position 351 was a histidine in A1 and an arginine in A3 (Figure 5). Swapping of this single amino acid difference between the domains (i.e., mutating the H to R in A1 and R to H in A3) transferred both pAg binding ability and functional ability to stimulate Vγ9Vδ2 T cells. Position 351 is quite buried within the pAg binding pocket; it is likely that the size and shape of the side chain difference from an H to an R changes the architecture of the binding pocket such the pAgs thus characterized do not bind to A3. This raises the possibility that there are other pyrophosphate compounds yet to be described that may preferentially bind to A3 over A1. It is unclear as to the role, if any, of the A2 and A3 isoforms in Vγ9Vδ2 T cell activation; their potential ability to form heterodimers with A1, assemble with alternative antigens via their B30.2 domain (in the case of A3) and modulate cell-surface assembly with other protein binding partners is an area of active investigation.

Additional insight into the mechanics of pAg binding to the B30.2 domain, pursued through crystallization and NMR experiments, have revealed evidence for a conformational change induced in the B30.2 domain upon pAg binding. The first insight into this was during our pursuit of a complex structure between the B30.2 domain and pAg where we attempted to “soak” pAg into already existing crystals (33). (This is a common approach for studying small molecule binding sites in proteins.) While protein crystals appear solid, they contain a substantial amount of liquid that forms solvent channels between the protein molecules. Thus, small molecules (such as pAgs) can move freely in the crystal lattice and bind to their appropriate binding site in the protein as it is locked in the crystal lattice. This methodology assumes that binding of the small molecule does not induce changes in the conformation of the protein as this can disrupt the packing of the protein in the crystal lattice and cause the crystals to dissolve. Soaking of B30.2 domain crystals with pAgs did just this, causing the crystals to dissolve immediately upon addition. Locking of the protein–protein contacts within the crystal lattice via covalent crosslinking via glutaraldehyde preserved the crystal structure and allowed a complex structure of pAg and B30.2 to be resolved. Within this structure there is clear tetrahedral electron density for the beta- and alpha- phosphates of the pAg within the binding pocket, however, the organic portion of the pAg could not be resolved (33). More direct evidence for a conformational change is seen in CSP observed in the Heteronuclear single quantum coherence spectroscopy (HSQC) of apo (empty) B30.2 versus that with added HMBPP (35). Thus, it is clear that binding of pAg to the B30.2 domain induces structural rearrangements/conformational changes that we hypothesize is the first in a cascade of intracellular and extracellular events leading to target cell transmission of a stimulatory signal to the Vγ9Vδ2 TCR.

This model of intracellular sensing of pAgs is consistent with the fact that many of the physiologically relevant pAgs are first generated and accumulate inside target cells. Endogenous pAgs, such as IPP or DMAPP, are intermediates of the MVA pathway, which is conserved in eukaryotes and archaea for isoprenoid biosynthesis (38). It has been reported that these pAgs accumulate intracellularly during dysregulated metabolism in many types of human tumor cells. For example, overexpression of HMG-CoA reductase, the rate limiting enzyme of the MVA pathway, in the non-Hodgkin B cell lymphoma cell-line Daudi and mammary cancer cells such as breast adenocarcinoma cells, can cause an increased level of IPP that is then recognized by Vγ9Vδ2 T cells (12, 39). Manipulation of the MVA pathway by various synthetic drugs (such as statins and aminobisphosphates) or short hairpin RNAs targeted to enzymes either upstream or downstream of IPP production can trigger or suppress pAg-induced T cell activation, supporting the idea that Vγ9Vδ2 T cells can sense intracellular IPP levels (12, 20, 21, 40). Another set of pAgs derive from exogenous microbial sources and are also much more potent. These metabolites, such as HMBPP from the microbial methylerythritol phosphate (MEP) pathway, exist in bacteria and several photosynthetic eukaryotes (38). Some microbes producing these pAgs are intracellular pathogens such as *Mycobacterium tuberculosis* and *Listeria monocytogenes*, which can enter and survive within the host cells (41, 42). Immune cells such as monocytes, macrophages and dendritic cells can also engulf these pathogens and elicit pAg-dependent T cell responses (43–46). Extracellular pathogens like *Escherichia coli* can also be phagocytosed by immune cells like neutrophils (47). The subsequent Vγ9Vδ2 T cell response is strictly dependent on the ability of the phagocytosed pathogens to produce HMBPP. A more recent study also demonstrated that after T cell priming, these pathogen-harboring neutrophils develop an antigen-presenting phenotype (48). These studies suggest that the presence of exogenous pAgs, *intracellularly*, is critical for Vγ9Vδ2 T cell activation and function. Admittedly, exogenous pAgs can also be secreted by some extracellular pathogens or immune cells like neutrophils, and administration of soluble pAgs in the presence of BTN3A-expressing antigen-presenting cells trigger Vγ9Vδ2 T cell activation, as was demonstrated in early studies of these T cells (24). It is unknown how these extracellular pAgs get internalized, giving that their negatively charged features render direct membrane permeability unlikely. Possible mechanisms for passing through the plasma and endocytic membranes may include specific membrane transporters or charge neutralization by ester formation.

## Phosphoantigen Structure and Bioactivity

The variable chemical structures of pAgs relate directly to their bioactivity. The pyrophosphate moiety is central since its strong negative charge can make electrostatic contacts with the positively charged binding pocket of the B30.2 domain. Indeed, monophosphate substituents have significantly reduced specific activity compared with their pyrophosphate counterparts (49). On the other hand, large chemical groups like AMP can be added without affecting the bioactivity of pAgs (49) and, in fact, some natural pAgs are nucleotidic conjugates (50, 51) that may be processed by specific antigen-presenting cells. More intriguingly, it has been found that hydrolysis of the pyrophosphate moiety is associated with pAg bioactivity and non-hydrolyzable analogs of pAgs can even inhibit the T cell activation (52). These features suggest that either pre- or post-processing of pAgs can occur before or after association with the B30.2 domain and may have important implications for T cell activation.

Since the pyrophosphate moiety is essentially the same for every pAg, the potency of pAgs is largely dependent on their organic moieties. Changes in the length of the alkyl chain and positions of the double bond, even though very subtle, can lead to dramatic change in potency (49). Notably, the structural difference between endogenous ligand IPP and exogenous HMBPP only lies in the additional hydroxyl group on HMBPP, yet the potency and binding affinity of this strong ligand increases by about 1000-fold. One possible explanation that was explored early on was the chemical reactivity of pAgs. The polarizability of the C3 substituent correlates with bioactivity: an increased specific activity for T cells was observed upon addition of a halohydrin to the C3 position (52). This leads to an interesting speculation that a covalent reaction may occur between the pAg and its binding partner. More recently, some studies have pointed out that the chirality of the pAgs may also play a role in their bioactivity. It has been found that E-stereoisomers are much more potent antigens for T cell activation (53). Potential intramolecular hydrogen-bonding states of these isomers affect the overall shape of the ligand and may be important for binding to the B30.2 domain. Most of the studies regarding the structure of pAgs were conducted before the identification of the B30.2 domain of BTN3A1 as the binding partner; optimization of binding to this domain has already begun with exciting implications for pAg-based therapeutics (35).

## Phosphoantigens Signal through an Inside-Out Mechanism via the B30.2 Domain

**Figure 8:**
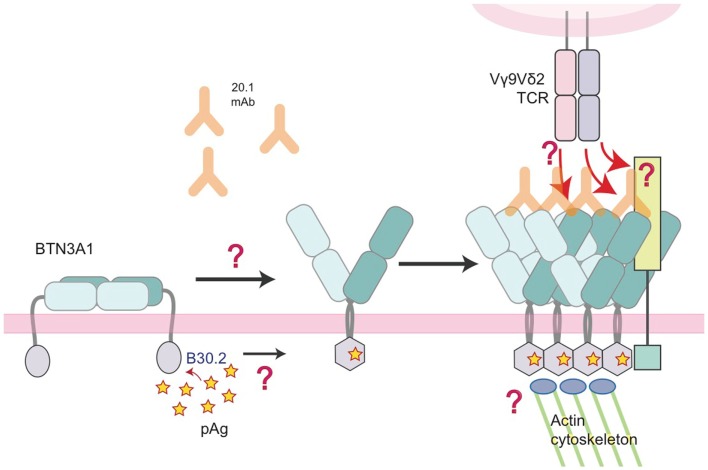
**Model of the molecular changes that occur in BTN3A molecules upon addition of 20.1 antibody or detection of accumulating intracellular pAg**. In this model, we propose that BTN3A molecules exist on the surface of a healthy, unaffected cell in an inactive state, perhaps in a conformation similar to the dimer 2 visualized in the crystal lattice (left). Upon addition of the 20.1 antibody, dimer 2 is destabilized and BTN3A molecules are converted/stabilized in the dimer 1 conformation. An increase in the intracellular pAg concentration has a similar effect except the dimer conversion is mediated by changes in the intracellular B30.2 domain, which undergoes a conformational change upon pAg binding (hexagonal shape). This conformational change induces structural reorganization of BTN3A molecules, either via immobilization through B30.2 association with the actin cytoskeleton or B30.2 multimerization of BTN3A molecules, which is then signaled by an inside-out mechanism to change the architecture of the BTN3A extracellular domains. This architectural change could alone be the signal that Vγ9Vδ2 TCRs recognize, or recruitment of an additional, human-specific accessory molecule (lime-green square) occurs, which directly engages the Vγ9Vδ2 TCR.

Of course, the detection of pAgs intracellularly needs to translate to a signal that can be detected by surveying Vγ9Vδ2 T cells. We and others have proposed an inside-out signaling model (31, 33) whereby the binding of pAg intracellularly is translated extracellularly for detection by the Vγ9Vδ2 TCR. This could be through several means that work individually or in concert to initiate TCR recognition: (1) immobilization/clustering of BTN3A that increases the avidity for the TCR, (2) a conformational change of the BTN3A extracellular domains from non-stimulatory (Dimer 2) to stimulatory (Dimer 1), or (3) the two previous situations resulting in the recruitment of an additional factor that directly engages the Vγ9Vδ2 TCR. Number 1 and 2 invoke a direct interaction between Vγ9Vδ2 TCRs and the extracellular domains of BTN3A whereas version 3 involves an unknown accessory protein that is the true Vγ9Vδ2 TCR ligand (Figure 8). Whether BTN3A is directly recognized by the Vγ9Vδ2 TCR is controversial; Vavassori and colleagues were able to measure an interaction between the IgV domain of BTN3A1 and a Vγ9Vδ2 TCR used in their studies (32) while we have not be able to do the same with the G115 Vγ9Vδ2 TCR and the full-length extracellular domain of BTN3A1 (33). In addition, we have not been able to stimulate Vγ9Vδ2 T cells with murine transfected BTN3A1 either through 20.1 antibody addition or through pAg treatment (33) whereas others, using a different Vγ9Vδ2 TCR have observed stimulation via 20.1 Ab treatment of BTN3A1 transfected Chinese-hamster ovary (CHO) cells (54). pAg-mediated stimulation was only observed in CHO cells containing human Chromosome 6. These conflicting results represent probably the most important conundrum in the Vγ9Vδ2 T cell field at present. BTN3A molecules are necessary but are they sufficient for Vγ9Vδ2 recognition? Evidence for other molecules playing a role in Vγ9Vδ2 recognition and activation such as HSP-60 (55) and F1-ATPase with or without ApoA1 (56) suggest this system could be a complicated coordination of many molecular players. Further coordination through adhesion molecules, activating and inhibitory Natural Killer receptors, Toll-like receptors (TLRs), and Fc Receptors [reviewed in Ref. (57)] also fine-tune Vγ9Vδ2 activation thresholds and functional responses.

A feature of Vγ9Vδ2 TCRs that may be important in answering the question regarding a direct interaction between the TCR and BTN3A is that despite the fact that this population uniformly use a Vγ9 and a Vδ2 chains in their TCR, there exists considerable diversity within the Vγ-Jγ and Vδ-Dδ-Jδ rearrangement, which translates into significant amino acid diversity within the CDR3γ and CDR3δ loops of these TCRs (58–60). These loops have been shown via mutagenesis to be important for Vγ9Vδ2 T cell activation (60) and sequence variation of these loops between T cell clones (controlled for expression of Natural Killer receptors) translates into a range of different, graded, reactivity (61). It is thus possible that use of Vγ9Vδ2 TCRs with different sequences, and thus different affinities for BTN3A, may be playing a role in the differing abilities to measure interactions between the Vγ9Vδ2 TCR and BTN3A. Alternatively there may be a requirement for additional co-factors to be expressed on target cells in conjunction with BTN3A to fully engage the majority of the Vγ9Vδ2 T cell repertoire.

Clearly, we have just begun to pull the curtain back on the inner-workings of Vγ9Vδ2 T cell ligand recognition and activation; identification of the B30.2 domain as the pAg sensor has already resulted in compounds developed with higher potency *in vitro* (35). Identification of additional players in this process will lead to important therapeutic targets with implications both for the treatment of tumors and microbial infections, but also for minimizing immune-related side effects during the use of bisphosphonates for the treatment for osteoporosis and cancer-related bone fractures.

## Conflict of Interest Statement

The authors declare that the research was conducted in the absence of any commercial or financial relationships that could be construed as a potential conflict of interest.

## References

[1] Salio M Silk JD Jones EY Cerundolo V. Biology of CD1- and MR1-restricted T cells. Annu Rev Immunol (2014) 32:323–66. 10.1146/annurev-immunol-032713-120243 24499274

[2] Born WK Kemal Aydintug M O’brien RL Diversity of gammadelta T-cell antigens. Cell Mol Immunol (2013) 10:13–20 10.1038/cmi.2012.45 23085946 PMC4003174

[3] Chien YH Meyer C Bonneville M. Gammadelta T cells: first line of defense and beyond. Annu Rev Immunol (2014) 32:121–55. 10.1146/annurev-immunol-032713-120216 24387714

[4] Kisielow J Kopf M. The origin and fate of gammadelta T cell subsets. Curr Opin Immunol (2013) 25:181–8. 10.1016/j.coi.2013.03.002 23562386

[5] Vantourout P Hayday A. Six-of-the-best: unique contributions of gammadelta T cells to immunology. Nat Rev Immunol (2013) 13:88–100. 10.1038/nri3384 23348415 PMC3951794

[6] Sturm E Bontrop RE Vreugdenhil RJ Otting N Bolhuis RL. T-cell receptor gamma/delta: comparison of gene configurations and function between humans and chimpanzees. Immunogenetics (1992) 36:294–301. 10.1007/BF00215657 1322863

[7] Kazen AR Adams EJ. Evolution of the V, D, and J gene segments used in the primate gammadelta T-cell receptor reveals a dichotomy of conservation and diversity. Proc Natl Acad Sci U S A (2011) 108:E332–40. 10.1073/pnas.1105105108 21730193 PMC3141992

[8] Luoma AM Castro CD Adams EJ. Gammadelta T cell surveillance via CD1 molecules. Trends Immunol (2014) 35(12):613–21. 10.1016/j.it.2014.09.003 25283967 PMC4383740

[9] Morita CT Mariuzza RA Brenner MB Antigen recognition by human gamma delta T cells: pattern recognition by the adaptive immune system. Springer Semin Immunopathol (2000) 22:191–217 10.1007/s002810000042 11116953

[10] Chen ZW. Multifunctional immune responses of HMBPP-specific Vgamma2Vdelta2 T cells in M. tuberculosis and other infections. Cell Mol Immunol (2013) 10:58–64. 10.1038/cmi.2012.46 23147720 PMC3664056

[11] Tanaka Y Sano S Nieves E De Libero G Rosa D Modlin RL Nonpeptide ligands for human gamma delta T cells. Proc Natl Acad Sci USA (1994) 91:8175–9. 10.1073/pnas.91.17.8175 8058775 PMC44568

[12] Gober HJ Kistowska M Angman L Jeno P Mori L De Libero G. Human T cell receptor gammadelta cells recognize endogenous mevalonate metabolites in tumor cells. J Exp Med (2003) 197:163–8. 10.1084/jem.20021500 12538656 PMC2193814

[13] Karunakaran MM Gobel TW Starick L Walter L Herrmann T. Vgamma9 and Vdelta2 T cell antigen receptor genes and butyrophilin 3 (BTN3) emerged with placental mammals and are concomitantly preserved in selected species like alpaca (*Vicugna pacos*). Immunogenetics (2014) 66:243–54. 10.1007/s00251-014-0763-8 24526346

[14] Morita CT Beckman EM Bukowski JF Tanaka Y Band H Bloom BR Direct presentation of nonpeptide prenyl pyrophosphate antigens to human gamma delta T cells. Immunity (1995) 3:495–507. 10.1016/1074-7613(95)90178-7 7584140

[15] Constant P Davodeau F Peyrat MA Poquet Y Puzo G Bonneville M Stimulation of human gamma delta T cells by nonpeptidic mycobacterial ligands. Science (1994) 264:267–70. 10.1126/science.8146660 8146660

[16] Tanaka Y Morita CT Nieves E Brenner MB Bloom BR. Natural and synthetic non-peptide antigens recognized by human gamma delta T cells. Nature (1995) 375:155–8. 10.1038/375155a0 7753173

[17] Hintz M Reichenberg A Altincicek B Bahr U Gschwind RM Kollas AK Identification of (E)-4-hydroxy-3-methyl-but-2-enyl pyrophosphate as a major activator for human gammadelta T cells in *Escherichia coli*. FEBS Lett (2001) 509:317–22. 10.1016/S0014-5793(01)03191-X 11741609

[18] Puan KJ Jin C Wang H Sarikonda G Raker AM Lee HK Preferential recognition of a microbial metabolite by human Vgamma2Vdelta2 T cells. Int Immunol (2007) 19:657–73. 10.1093/intimm/dxm031 17446209

[19] Burk MR Mori L De Libero G. Human V gamma 9-V delta 2 cells are stimulated in a cross-reactive fashion by a variety of phosphorylated metabolites. Eur J Immunol (1995) 25:2052–8. 10.1002/eji.1830250737 7621879

[20] Kunzmann V Bauer E Wilhelm M Gamma/delta T-cell stimulation by pamidronate. N Engl J Med (1999) 340:737–8 10.1056/NEJM199903043400914 10068336

[21] Thompson K Rojas-Navea J Rogers MJ. Alkylamines cause Vgamma9Vdelta2 T-cell activation and proliferation by inhibiting the mevalonate pathway. Blood (2006) 107:651–4. 10.1182/blood-2005-03-1025 16179378

[22] Espinosa E Belmant C Pont F Luciani B Poupot R Romagne F Chemical synthesis and biological activity of bromohydrin pyrophosphate, a potent stimulator of human gamma delta T cells. J Biol Chem (2001) 276:18337–44. 10.1074/jbc.M100495200 11279081

[23] Bukowski JF Morita CT Tanaka Y Bloom BR Brenner MB Band H. V gamma 2V delta 2 TCR-dependent recognition of non-peptide antigens and Daudi cells analyzed by TCR gene transfer. J Immunol (1995) 154:998–1006. 7529807

[24] Lang F Peyrat MA Constant P Davodeau F David-Ameline J Poquet Y Early activation of human V gamma 9V delta 2 T cell broad cytotoxicity and TNF production by nonpeptidic mycobacterial ligands. J Immunol (1995) 154:5986–94. 7751641

[25] Harly C Guillaume Y Nedellec S Peigne CM Monkkonen H Monkkonen J Key implication of CD277/butyrophilin-3 (BTN3A) in cellular stress sensing by a major human gammadelta T-cell subset. Blood (2012) 120:2269–79. 10.1182/blood-2012-05-430470 22767497 PMC3679641

[26] Rhodes DA Stammers M Malcherek G Beck S Trowsdale J. The cluster of BTN genes in the extended major histocompatibility complex. Genomics (2001) 71:351–62. 10.1006/geno.2000.6406 11170752

[27] Palakodeti A Sandstrom A Sundaresan L Harly C Nedellec S Olive D The molecular basis for modulation of human Vgamma9Vdelta2 T cell responses by CD277/butyrophilin-3 (BTN3A)-specific antibodies. J Biol Chem (2012) 287:32780–90. 10.1074/jbc.M112.384354 22846996 PMC3463320

[28] Abeler-Dorner L Swamy M Williams G Hayday AC Bas A. Butyrophilins: an emerging family of immune regulators. Trends Immunol (2012) 33:34–41. 10.1016/j.it.2011.09.007 22030238

[29] Arnett HA Viney JL Immune modulation by butyrophilins. Nat Rev Immunol (2014) 14:559–69 10.1038/nri3715 25060581

[30] Decaup E Duault C Bezombes C Poupot M Savina A Olive D Phosphoantigens and butyrophilin 3A1 induce similar intracellular activation signaling in human TCRVgamma9+ gammadelta T lymphocytes. Immunol Lett (2014) 161:133–7. 10.1016/j.imlet.2014.05.011 24925024

[31] Wang H Henry O Distefano MD Wang YC Raikkonen J Monkkonen J Butyrophilin 3A1 plays an essential role in prenyl pyrophosphate stimulation of human Vgamma2Vdelta2 T cells. J Immunol (2013) 191:1029–42. 10.4049/jimmunol.1300658 23833237 PMC3884521

[32] Vavassori S Kumar A Wan GS Ramanjaneyulu GS Cavallari M El Daker S Butyrophilin 3A1 binds phosphorylated antigens and stimulates human gammadelta T cells. Nat Immunol (2013) 14:908–16. 10.1038/ni.2665 23872678

[33] Sandstrom A Peigne CM Leger A Crooks JE Konczak F Gesnel MC The intracellular B30.2 domain of butyrophilin 3A1 binds phosphoantigens to mediate activation of human Vgamma9Vdelta2 T cells. Immunity (2014) 40:490–500. 10.1016/j.immuni.2014.03.003 24703779 PMC4028361

[34] Rhodes DA De Bono B Trowsdale J. Relationship between SPRY and B30.2 protein domains. Evolution of a component of immune defence? Immunology (2005) 116:411–7. 10.1111/j.1365-2567.2005.02248.x 16313355 PMC1802431

[35] Hsiao CH Lin X Barney RJ Shippy RR Li J Vinogradova O Synthesis of a phosphoantigen prodrug that potently activates Vgamma9Vdelta2 T-lymphocytes. Chem Biol (2014) 21:945–54. 10.1016/j.chembiol.2014.06.006 25065532

[36] Altincicek B Moll J Campos N Foerster G Beck E Hoeffler JF Cutting edge: human gamma delta T cells are activated by intermediates of the 2-C-methyl-D-erythritol 4-phosphate pathway of isoprenoid biosynthesis. J Immunol (2001) 166:3655–8. 10.4049/jimmunol.166.6.3655 11238603

[37] James LC Keeble AH Khan Z Rhodes DA Trowsdale J. Structural basis for PRYSPRY-mediated tripartite motif (TRIM) protein function. Proc Natl Acad Sci U S A (2007) 104:6200–5. 10.1073/pnas.0609174104 17400754 PMC1851072

[38] Lombard J Moreira D. Origins and early evolution of the mevalonate pathway of isoprenoid biosynthesis in the three domains of life. Mol Biol Evol (2011) 28:87–99. 10.1093/molbev/msq177 20651049

[39] Asslan R Pradines A Pratx C Allal C Favre G Le Gaillard F. Epidermal growth factor stimulates 3-hydroxy-3-methylglutaryl-coenzyme A reductase expression via the ErbB-2 pathway in human breast adenocarcinoma cells. Biochem Biophys Res Commun (1999) 260:699–706. 10.1006/bbrc.1999.0945 10403829

[40] Li J Herold MJ Kimmel B Muller I Rincon-Orozco B Kunzmann V Reduced expression of the mevalonate pathway enzyme farnesyl pyrophosphate synthase unveils recognition of tumor cells by Vgamma9Vdelta2 T cells. J Immunol (2009) 182:8118–24. 10.4049/jimmunol.0900101 19494338

[41] Eberl M Hintz M Reichenberg A Kollas AK Wiesner J Jomaa H. Microbial isoprenoid biosynthesis and human gammadelta T cell activation. FEBS Lett (2003) 544:4–10. 10.1016/S0014-5793(03)00483-6 12782281

[42] Silva MT. Classical labeling of bacterial pathogens according to their lifestyle in the host: inconsistencies and alternatives. Front Microbiol (2012) 3:71. 10.3389/fmicb.2012.00071 22393329 PMC3289908

[43] Dieli F Troye-Blomberg M Ivanyi J Fournie JJ Bonneville M Peyrat MA Vgamma9/Vdelta2 T lymphocytes reduce the viability of intracellular *Mycobacterium tuberculosis*. Eur J Immunol (2000) 30:1512–9. 10.1002/(SICI)1521-4141(200005)30:5<1512::AID-IMMU1512>3.0.CO;2-3 10820400

[44] Rojas RE Torres M Fournie JJ Harding CV Boom WH. Phosphoantigen presentation by macrophages to *Mycobacterium tuberculosis* – reactive Vgamma9Vdelta2+ T cells: modulation by chloroquine. Infect Immun (2002) 70:4019–27. 10.1128/IAI.70.8.4019-4027.2002 12117907 PMC128132

[45] Devilder MC Maillet S Bouyge-Moreau I Donnadieu E Bonneville M Scotet E. Potentiation of antigen-stimulated V gamma 9V delta 2 T cell cytokine production by immature dendritic cells (DC) and reciprocal effect on DC maturation. J Immunol (2006) 176:1386–93. 10.4049/jimmunol.176.3.1386 16424165

[46] Wei H Huang D Lai X Chen M Zhong W Wang R Definition of APC presentation of phosphoantigen (E)-4-hydroxy-3-methyl-but-2-enyl pyrophosphate to Vgamma2Vdelta 2 TCR. J Immunol (2008) 181:4798–806. 10.4049/jimmunol.181.7.4798 18802083 PMC2743079

[47] Davey MS Lin CY Roberts GW Heuston S Brown AC Chess JA Human neutrophil clearance of bacterial pathogens triggers anti-microbial gammadelta T cell responses in early infection. PLoS Pathog (2011) 7:e1002040. 10.1371/journal.ppat.1002040 21589907 PMC3093373

[48] Davey MS Morgan MP Liuzzi AR Tyler CJ Khan MW Szakmany T Microbe-specific unconventional T cells induce human neutrophil differentiation into antigen cross-presenting cells. J Immunol (2014) 193:3704–16. 10.4049/jimmunol.1401018 25165152 PMC4169984

[49] Morita CT Lee HK Wang H Li H Mariuzza RA Tanaka Y. Structural features of nonpeptide prenyl pyrophosphates that determine their antigenicity for human gamma delta T cells. J Immunol (2001) 167:36–41. 10.4049/jimmunol.167.1.36 11418629

[50] Poquet Y Constant P Halary F Peyrat MA Gilleron M Davodeau F A novel nucleotide-containing antigen for human blood gamma delta T lymphocytes. Eur J Immunol (1996) 26:2344–9. 10.1002/eji.1830261011 8898943

[51] Vantourout P Martinez LO Fabre A Collet X Champagne E. Ecto-F1-ATPase and MHC-class I close association on cell membranes. Mol Immunol (2008) 45:485–92. 10.1016/j.molimm.2007.05.026 17643490

[52] Belmant C Espinosa E Halary F Tang Y Peyrat MA Sicard H A chemical basis for selective recognition of nonpeptide antigens by human delta T cells. FASEB J (2000) 14:1669–70 10.1096/fj.99-0909fje 10973912

[53] Boedec A Sicard H Dessolin J Herbette G Ingoure S Raymond C Synthesis and biological activity of phosphonate analogues and geometric isomers of the highly potent phosphoantigen (E)-1-hydroxy-2-methylbut-2-enyl-4-diphosphate. J Med Chem (2008) 51:1747–54. 10.1021/jm701101g 18303828

[54] Riano F Karunakaran MM Starick L Li J Scholz CJ Kunzmann V Vgamma9Vdelta2 TCR-activation by phosphorylated antigens requires butyrophilin 3 A1 (BTN3A1) and additional genes on human chromosome 6. Eur J Immunol (2014) 44:2571–6. 10.1002/eji.201444712 24890657

[55] Kaur I Voss SD Gupta RS Schell K Fisch P Sondel PM. Human peripheral gamma delta T cells recognize hsp60 molecules on Daudi Burkitt’s lymphoma cells. J Immunol (1993) 150:2046–55. 8094731

[56] Scotet E Martinez LO Grant E Barbaras R Jeno P Guiraud M Tumor recognition following Vgamma9Vdelta2 T cell receptor interactions with a surface F1-ATPase-related structure and apolipoprotein A-I. Immunity (2005) 22:71–80. 10.1016/j.immuni.2004.11.012 15664160

[57] Nedellec S Bonneville M Scotet E. Human Vgamma9Vdelta2 T cells: from signals to functions. Semin Immunol (2010) 22:199–206. 10.1016/j.smim.2010.04.004 20447835

[58] Bukowski JF Morita CT Band H Brenner MB. Crucial role of TCR gamma chain junctional region in prenyl pyrophosphate antigen recognition by gamma delta T cells. J Immunol (1998) 161:286–93. 9647235

[59] Miyagawa F Tanaka Y Yamashita S Mikami B Danno K Uehara M Essential contribution of germline-encoded lysine residues in Jgamma1.2 segment to the recognition of nonpeptide antigens by human gammadelta T cells. J Immunol (2001) 167:6773–9. 10.4049/jimmunol.167.12.6773 11739492

[60] Wang H Fang Z Morita CT. Vgamma2Vdelta2 T Cell receptor recognition of prenyl pyrophosphates is dependent on all CDRs. J Immunol (2010) 184:6209–22. 10.4049/jimmunol.1000231 20483784 PMC3069129

[61] Grunder C Van Dorp S Hol S Drent E Straetemans T Heijhuurs S Gamma9 and delta2CDR3 domains regulate functional avidity of T cells harboring gamma9delta2TCRs. Blood (2012) 120:5153–62. 10.1182/blood-2012-05-432427 23018643

